# Kampo medicine for palliative care in Japan

**DOI:** 10.1186/1751-0759-8-6

**Published:** 2014-01-22

**Authors:** Hirokuni Okumi, Atsuko Koyama

**Affiliations:** 1 Department of Clinical Oncology, Division of Psychosomatic Medicine, Kinki University, Faculty of Medicine, Osaka, Japan

**Keywords:** Kampo medicine, Palliative care, Cachexia, Chemotherapy

## Abstract

Kampo medicines are currently manufactured under strict quality controls. The Ministry of Health, Labour and Welfare of Japan has approved 148 Kampo formulas. There is increasing evidence for the efficacy of Kampo medicines, and some are used clinically for palliative care in Japan. The specific aim of this review is to evaluate the clinical use of Kampo medicines in palliative care in the treatment of cancer. The conclusions are as follows: Juzentaihoto inhibits the progression of liver tumors in a dose-dependent manner and contributes to long-term survival. Hochuekkito has clinical effects on cachexia for genitourinary cancer and improves the QOL and immunological status of weak patients, such as postoperative patients. Daikenchuto increases intestinal motility and decreases the postoperative symptoms of patients with total gastrectomy with jejunal pouch interposition, suppresses postoperative inflammation following surgery for colorectal cancer, and controls radiation-induced enteritis. Rikkunshito contributes to the amelioration of anorectic conditions in cancer cachexia-anorexia syndrome. Goshajinkigan and Shakuyakukanzoto reduce the neurotoxicity of patients with colorectal cancer who undergo oxaliplatin and FOLFOX (5-fluorouracil/folinic acid plus oxaliplatin) therapy. Hangeshashinto has the effect of preventing and alleviating diarrhea induced by CPT-11(irinotecan) and combination therapy with S-1/CPT-11. O’rengedokuto significantly improves mucositis caused by anticancer agents.

## Introduction

Traditional Japanese medicine, including the use of Kampo medicines, has been practiced for 1500 years. These formulas are intended not only to help recover the natural balance of the human body, but also treat the body’s impairments, such as indigestion and fatigue [1]. Kampo medicine is practiced widely and is integrated with Western medicine in Japan.

Iwase et al. [2] distributed a cross-sectional self-administered anonymous questionnaire to 549 physicians working in palliative care at 388 core cancer treatment hospitals and 161 certified medical institutions with palliative care units. In total, 311 physicians responded. They were evenly distributed throughout the country without significant geographical biases. Kampo medicines were prescribed for controlling cancer-related symptoms by 64.3% of the physicians. The symptoms treated with Kampo medicines were numbness/hypoesthesia (49.5%), constipation (38.0%), anorexia/weight loss (36%), muscle cramps (35.5%), and languor/fatigue (32.0%). With regard to issues about prescription, 60.7% of the physicians raised the issue that the dosage forms need to be better devised.

Ito et al. [3] reported that, among 900 physicians surveyed, 92.4% reported having prescribed Kampo medications, 73.5% of whom reported having prescribed them for cancer patients. Despite this high percentage and that only 9.7% of the physicians reported that they considered Kampo medications to be harmful, only 23.1% of the physicians expressed high expectations of the efficacy of Kampo medicine in tumor suppression and the exertion of immunostimulatory action.

The purpose of this paper is to investigate the clinical use of Kampo for patients who need palliative care. Therefore, we conducted a literature survey of current reports on Kampo medicine use in cancer-related treatments.

## Review

### Prevention of malignant progression and metastasis (Juzentaihoto, Hochuekkito)

The traditional Japanese medicine Juzentaihoto is a pharmaceutical grade multi-herbal medicine used for the activation of hematopoiesis and reduction of side effects from chemotherapy and radiotherapy.

Ohnishi et al. [4] reported that oral administration of Juzentaihoto for 7 days before tumor inoculation resulted in a dose-dependent inhibition of liver tumor colonies and a significantly higher survival rate as compared with the untreated control, and without side effects. Additionally natural killer cells, macrophages, and T-cells play important roles in the prevention of metastasis of tumor cells and activated peritoneal exudate macrophages to become cytostatic against the tumor cells in the host immune system. It is suggested that Juzentaihoto inhibits the progression of liver tumor in a dose-dependent manner and that it contributes to long-term survival.

Hochuekkito is a Kampo formula composed of ten crude drugs from plants. It is used for the treatment of general fatigue caused by common colds or ordinary life. Hochuekkito improved the quality of life (QOL) and immunological status of elderly patients [5].

Kuroda et al. [6] performed a clinical study on 162 patients who complained of anorexia or lassitude because of genitourinary cancer. Each patient was administered Hochuekkito (7.5 g/day). The efficacy rate was 63.0%. The rate of effectiveness on anorexia was 48.4% and that on lassitude was 36.6%. Side effects were observed in 12 patients (7.4%), but most were mild gastrointestinal disorders. No severe adverse effects were noted. This study indicates that Hochuekkito had clinical effects on cachexia for genitourinary cancer.

### Modulation of immunological factors and surgical stress (Hochuekkito, Daikenchuto)

Intestinal motility after gastrointestinal surgery to remove cancer frequently is disturbed and results in postoperative intestinal symptoms and poor QOL because gastrointestinal organs modulate the immunology of the human body.

The intestinal epithelial cells sit at the interface between the lumen and lamina propria or lymph nodes such as Peyer’s patches, where they maintain intestinal homeostasis through chemokine secretion.

Satoh et al. [7] evaluated the effects of Hochuekkito in the treatment of fifteen elderly patients with general weakness. A multicenter, prospective, randomized, double-blind, placebo-controlled study with an N of one and responder restricted design was performed. Only responders were randomized into three groups: an active-placebo group, a placebo-active group, and an active-active group. The study consisted of two 6-week terms with a 2-week washout period. The physical component summary from the Short Form 36 Health Survey (SF-36) analysis was significantly improved in the Hochuekkito-treated group. Four of six components (A-H: anger-hostility, F: fatigue, T-A: tension-anxiety, C: confusion) were improved in the Hochuekkito-treated group in the Profile of Mood States (POMS) analysis. Lymphocyte proliferating activity improved in the Hochuekkito-treated group, but not significantly. Moreover, the population of CD3 positive cells and CD3CD4 double positive cells in the surface antigens of peripheral lymphocytes increased in the Hochuekkito-treated group. It was suggested that Hochuekkito improved the QOL and immunological status of patients with weaknesses, such as postoperative patients.

The inflammatory response after surgery is associated with various postoperative complications. Endo et al. [8] examined the effects of Daikenchuto on intestinal motility and the postoperative QOL of patients. Seventeen patients who underwent total gastrectomy with jejunal pouch interposition for gastric cancer were assigned randomly in the crossover study with or without Daikenchuto using ^111^In-labeled liquid and ^99m^Tc-labeled solid test meal. In addition, a manometric study was performed to measure contractile activity with or without Daikenchuto. Stasis-related symptoms were reduced significantly by Daikenchuto. In the emptying test, Daikenchuto accelerated the emptying of both liquid and solid meals from the pouch. The pouch showed bursts of contractions, which were increased significantly by the oral intake of Daikenchuto. Daikenchuto increased intestinal motility and decreased the postoperative symptoms of patients with total gastrectomy with jejunal pouch interposition.

Yoshikawa et al. [9] reported the effects of Daikenchuto on the inflammatory response of patients following laparoscopic colorectal resection. Thirty patients who underwent laparoscopic colectomy for colorectal carcinoma were divided into a Daikenchuto group (D group, 7.5 g/day from the day after surgery until the seventh postoperative day) and a control group (C group). The time until first flatus was significantly shorter in the D group than in the C group. The C reactive protein (CRP) level was significantly lower in the D group than in the C group on the third postoperative day. Daikenchuto administration significantly suppressed postoperative inflammation following surgery for colorectal cancer.

Radiation-induced enteritis is a serious clinical problem for which there is no current standard management. Takeda et al. [10] evaluated a patient with radiation-induced enteritis whose clinical symptoms were much improved by treatment with Daikenchuto administered orally (7.5 g/day). Abdominal distention was evaluated objectively with computed tomography. Gastrointestinal symptoms associated with radiation-induced enteritis were controlled successfully with Daikenchuto. Daikenchuto treatment may be useful for the management of radiation-induced enteritis, according to this case study.

### Nutritional support for malnourished cancer patients (Rikkunshito)

Cachexia, a major cause of cancer-related death, is characterized by depletion of muscle and fat tissues, anorexia, asthenia, and hypoglycemia. Cachexic patients have deranged carbohydrate, lipid, and protein metabolism, induced by inflammatory cytokines.

Cheng et al. [11] reported which herbal approaches have had associated cancer cachexia case reports. These herbal medicines include *Panax ginsen*g, *Cimicifuga rhizome*, and *Radix astragali*, which are found in Juzentaihoto, Hochuekkito, O’gonto, and Rikkunshito.

Takeda et al. [12,13] evaluated the orexigenic effect of Rikkunshito with a focus on interaction with the ghrelin signaling system in cancer patients with chemotherapy-induced dyspepsia. Oral administration of Rikkunshito potentiated the orexigenic action of ghrelin through several different mechanisms. Human studies indicate that Rikkunshito is a promising therapeutic option for anorectic conditions, including cancer cachexia-anorexia syndrome.

### Prevention of side effects (peripheral neuropathy, allodynia, hyperalgesia) of paclitaxel (Goshajinkigan)

Paclitaxel is used for the treatment of solid tumors such as those in breast, ovarian, and lung cancer. However, it can induce peripheral neuropathy and severe muscle dysfunction. Carboplatin**/**paclitaxel chemotherapy for cancer (TC therapy) exhibits neurotoxicity and commonly causes peripheral neuropathy, which is difficult to cope with.

Kaku et al. [14] reported the efficacy of Goshajinkigan for patients with ovarian or endometrial cancer who underwent TC therapy and developed peripheral neuropathy. Patients were randomly divided into Group A, with 14 patients (vitamin B12 treatment), and Group B, with 15 patients (vitamin B12 + Goshajinkigan treatment). The observation period was 6 weeks following treatment initiation, and the evaluation items were as follows: i) current perception threshold of the peripheral nerve, ii) visual analogue scale for numbness, iii) grade of neurotoxicity, and iv) subjective symptoms of peripheral neuropathy. Items were compared between the groups and no significant differences were noted in any item. However, neurotoxicity developed in some patients Group A after 6 weeks, whereas no neurotoxicity was observed in Group B. Moreover, the frequency of abnormal values was significantly lower in Group B than in Group A. Therefore, Goshajinkigan may inhibit the progression of peripheral neuropathy after chemotherapy.

### Prevention of a peripheral neuropathy from oxaliplatin (Goshajinkigan, Shakuyaku- kanzoto)

Oxaliplatin is used in the treatment of colorectal cancer, but it causes acute and chronic neuropathies. Peripheral neurotoxicity is the major limiting factor for oxaliplatin therapy. Goshajinkigan is a Kampo medicine used for the treatment of several neurological symptoms, including pain and numbness. More recently, the formula has been reported to clinically prevent oxaliplatin-induced peripheral neuropathy.

A combination of 5-fluorouracil/folinic acid plus oxaliplatin (FOLFOX) is a standard regimen for the chemotherapy of metastatic colorectal cancer. The major dose-limiting toxic effect of oxaliplatin is neurotoxicity. Kono et al. [15] retrospectively investigated the effect of Goshajinkigan on peripheral neurotoxicity associated with oxaliplatin therapy. Ninety patients with metastatic colorectal cancer who received FOLFOX6 therapy were assigned to receive one of the following adjuncts: oral Goshajinkigan (7.5 g/day) (Group A), intravenous supplementation of calcium gluconate and magnesium sulfate (1 g each before and after FOLFOX) (Group B), Goshajinnkigan, calcium gluconate, and magnesium sulfate therapies (Group C), or no concomitant therapy (Group D). The incidence of peripheral neurotoxicity was investigated when the cumulative dose of oxaliplatin exceeded 500 mg/m^2^. When the cumulative dose of oxaliplatin exceeded 500 mg/m^2^, the incidence of neuropathy (all grades) in Groups A–D was 50.0, 100, 78.9, and 91.7%, respectively. It was lowest in the group that received Goshajinkigan alone. Concomitant administration of Goshajinkigan reduced the neurotoxicity of oxaliplatin in patients who received chemotherapy for colorectal cancer.

Hosokawa et al. [16] evaluated the preventive effects of Goshajinkigan and Shakuyaku- kanzoto on oxaliplatin-induced neurotoxicity with FOLFOX. Patients with metastatic colorectal cancer who received modified FOLFOX6 or FOLFOX4 for three years received either Goshajinkigan (group A) or Shakuyakukanzoto (group B). The response rate of the 38 patients with measurable lesions was 50.0% (9/18) in group A and 65% (13/20) in group B, in the cumulative dose exceeding 500 mg/m^2^. The administration of traditional Japanese medicine may reduce oxaliplatin-induced neurotoxicity without negatively affecting tumor response in patients with colorectal cancer who undergo FOLFOX therapy.

### Prevention of diarrhea from irinotecan (Hangeshashinto)

The chemotherapeutic agent CPT-11 (irinotecan) has shown promising results as a single agent and in combination chemotherapy for the treatment of colorectal and small cell lung cancer. Preventing CPT-11-induced delayed diarrhea, oral alkalization, and control of defecation has been studied. Oral administration of antibiotics or Kampo medicine to decrease beta-glucuronidase activity derived from bacteria in the large intestine was reported to be successful in preventing delayed diarrhea. When CPT-11-induced delayed diarrhea occurs, the conventional treatment is loperamide [17].

Mori K et al. [18] conducted a randomized comparative trial of 41 previously untreated patients with advanced non-small-cell lung cancer to investigate whether support with Hangeshashinto would prevent and control CPT-11-induced diarrhea. The chemotherapy regimen consisted of a combination of cisplatin and CPT-11. TJ-14 (7.5 g/day) was administered orally. Compared with the control group, although no differences in the frequency of diarrhea or the number of days the symptoms continued, the Hangeshashinto group showed a significant improvement in diarrhea grades (P = 0.044) as well as a reduced frequency of diarrhea grades 3 and 4 (one patient versus ten patients; P = 0.018).

It is suggested that Hangeshashinto, which contains baicalin, a beta-glucuronidase inhibitor, alleviates diarrhea induced by CPT-11.

Hibi et al. [19] prospectively investigated the influence of Hangeshashinto on the therapeutic and adverse effects of chemotherapy and the changes in QOL scores of patients with metastatic gastric and colorectal cancer from 2007 to 2008. Twenty patients receiving S-1/CPT-11 therapy were randomly allocated into group A (with Hangeshashinto) and B (control). While the anti-tumor effects did not differ significantly between these two groups, severe side effects of more than grade 3 occurred less frequently in group A. Moreover, the decrease in QOL scores on day 15 were improved in group A compared to group B. Therefore, Hangeshashinto could be used as a supportive medicine in the combination therapy of S-1/CPT-11.

### Prevention of other side effects from chemotherapy (O’rengedokuto)

Most anticancer agents cause xerostomia and mucositis, such as stomatitis and gastrointestinal mucosal injury, which is associated with infections, a decrease in QOL, and discontinuation of chemotherapy in patients with malignancy.

Yuki et al. [20] retrospectively evaluated the preventive effect of the oral administration of O’rengedokuto on stomatitis and diarrhea induced by cytotoxic drugs in 40 patients with acute leukemia. The incidence of stomatitis was 27.9% in the group given O’rengedokuto, which was significantly lower compared with 71.6% of those who received a gargle consisting of allopurinol, sodium gualenate, and povidone iodine. Drug-induced diarrhea was observed in 9.3% of the O’rengedokuto group compared with 31.7% of the control group. These observations indicate that O’rengedokuto significantly improved mucositis caused by anticancer agents.

### Others

Several reports have presented the efficacy of Kampo medicines on palliative care. Some clinical articles about Kampo medicine have reported the effect of Bakumondoto on dry cough and thirst, Hangekobokuto on dysphasia and depression, Kikyoto on stomatitis and pharyngeal pain involved in radiotherapy, and Yokukansan on delirium in palliative care.

Some basic research indicates that bone marrow suppression by treatment with TS-1 might be improved by the coadministration of Juzentaihoto or Ninjin’yoeito. Juzentaihoto also could be an effective drug for protecting against the side effects induced by cisplatin and carboplatin.

These formulas are known as “Hozai” or “Rikizai” and are used to activate hematopoiesis and treat anorexia in Japan, but there is a lack of the evidence for their effectiveness in clinical palliative care.

## Conclusions

Many palliative care physicians prescribed Kampo medicine because of the expectation that it would control the symptoms or adverse side effects of chemotherapy for cancer patients. The concepts on which Kampo (oriental) medicines are prescribed are much different from those of Western medicine. Physicians who specialize in Kampo medicine traditionally prescribe formulas based on the status of the physical and mental aspects of the patient (Sho). However, most palliative care physicians have no experience with Kampo medicine and must depend on evidence for drug efficacy. The prescription of Kampo formulas is limited because of the lack of evidence-based reports.

More evidence from clinical studies is desirable, and further research into the efficacy of Kampo medicine for cancer patients is warranted in Japan.

## Abbreviations

In: Indium; Tc: Technetium.

## Competing interests

The authors declare that they have no competing interests.

## Authors’ contributions

TO conceived and designed the study. OH collected previous reports of Kampo medicine regarding cancer-related palliative care and reviewed them. He categorized its usage into ten types and elaborated on them. AK had the responsibility for the final approval of the article. Both authors read and approved the final manuscript.
